# Phytoassisted synthesis of magnesium oxide nanoparticles from *Pterocarpus marsupium* rox.b heartwood extract and its biomedical applications

**DOI:** 10.1186/s43141-021-00119-0

**Published:** 2021-01-28

**Authors:** Manne Anupama Ammulu, K. Vinay Viswanath, Ajay Kumar Giduturi, Praveen Kumar Vemuri, Ushakiranmayi Mangamuri, Sudhakar Poda

**Affiliations:** 1 Department of Biotechnology, Acharya Nagarjuna University, Nagarjuna Nagar, Guntur, Andhra Pradesh 522510 India; 2 Freshman Engineering Department, PVP Siddhartha Institute of Technology, Chalasani Nagar, Kanuru, Vijayawada, Andhra Pradesh 520007 India; 3 Department of Biotechnology, Koneru Lakshmaiah Education Foundation, Guntur, Andhra Pradesh India; 4 Department of Botany and Microbiology, Acharya Nagarjuna University, Nagarjuna Nagar, Guntur, Andhra Pradesh 522510 India

**Keywords:** Magnesium oxide nanoparticles, *Pterocarpus marsupium*, Antioxidant activity, Antimicrobial activity, Anti-diabetic activity, Anti-inflammatory activity

## Abstract

**Background:**

Unlike chemical techniques, the combination of metal oxide nanoparticles utilizing plant concentrate is a promising choice. The purpose of this work was to synthesize magnesium oxide nanoparticles (MgO-NPs) utilizing heartwood aqueous extract of *Pterocarpus marsupium*. The heartwood extract of *Pterocarpus marsupium* is rich in polyphenolic compounds and flavonoids that can be used as a green source for large-scale, simple, and eco-friendly production of MgO-NPs. The phytoassisted synthesis of MgO is characterized by UV-Visible spectroscopy, X-ray diffraction (XRD), dynamic light scattering (DLS), Fourier transform infrared spectroscopy (FT-IR), scanning electron microscopy (SEM) with EDS (energy dispersive X-ray spectroscopy), and transmission electron microscopy (TEM).

**Results:**

The formation of MgO-NPs is confirmed by a visual color change from colorless to dark brown and they displayed a wavelength of 310 nm in UV-Spectrophotometry analysis. The crystalline nature of the obtained biosynthesized nanoparticles are revealed by X-ray diffraction analysis. SEM results revealed the synthesized magnesium oxide nanoparticles formed by this cost-effective method are spherically shaped with an average size of < 20 nm. The presence of magnesium and oxygen were confirmed by the EDS data. TEM analysis proved the spherical shape of the nanoparticles with average particle size of 13.28 nm and SAED analysis confirms the crystalline nature of MgO-NPs. FT-IR investigation confirms the existence of the active compounds required to stabilize the magnesium oxide nanoparticles with hydroxyl and carboxyl and phenolic groups that act as reducing, stabilizing, and capping agent. All the nanoparticles vary in particle sizes between 15 and 25 nm and obtained a polydispersity index value of 0.248. The zeta-potential was measured and found to be − 2.9 mV. Further, MgO-NPs were tested for antibacterial action against *Staphylococcus aureus* (Gram-positive bacteria) and *Escherichia coli* (Gram-negative bacteria) by minimum inhibitory concentration technique were found to be potent against both the bacteria. The blended nanoparticles showed good antioxidant activity examined by the DPPH radical scavenging method, showed good anti-diabetic activity determined by alpha-amylase inhibitory activity, and displayed strong anti-inflammatory activity evaluated by the albumin denaturation method.

**Conclusions:**

The investigation reports the eco-friendly, cost-effective method for synthesizing magnesium oxide nanoparticles from *Pterocarpus marsupium* Rox.b heartwood extract with biomedical applications.

**Graphical abstract:**

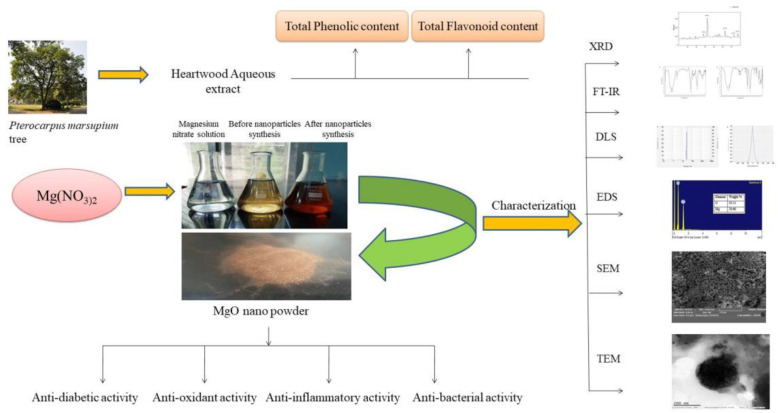

## Background

Conventional methods have been used for many years, but research has shown that green synthesis methods are more efficient in generating nanoparticles with less possibilities of failure, inexpensive, and ease of characterization [1, 2]. Due to their poisonous metabolites, physical and chemical methods to synthesize nanoparticles (NPs) have presented many stresses on the environment. Nanomaterial synthesis may be conducted using methods based on the template-assisted, vapor-liquid-solid, colloidal micellar, sol-gel, and microwave process. Magnesium oxide nanoparticles (MgO-NPs) are usually synthesized by various magnesium salts being decomposed thermally [3]. The latter methodologies are not preferred because of the extreme conditions of reaction, the use of poisonous reagents, expensive facilities, high pressure, and the usage of conservative non-renewable forms of energy [4]. However, MgO-NPs prepared using these conventional methods often contain a relatively small surface area and are therefore of low reaction rate [5]. In general, biochemicals like polysaccharides, enzymes, and vitamins found in micro-organisms such as bacteria [6], fungi [7], algae [8], and plants [9] could be utilized for the biosynthesis of nanoparticles [10, 11].

Plant-based NP synthesis is not a difficult process; a metal salt is synthesized with plant extract and the process is completed at normal room temperature in minutes to a couple of hours. Especially for silver (Ag) and gold (Au) NPs (nanoparticles), which are more stable when opposed to other metallic NPs, this technique has gained even more interest over the last decade. It can be effortlessly scaled up to generate NPs from green techniques and they are also fiscally smart. The greenly designed NPs are preferred over generally produced NPs in terms of their exceptional properties. The usage of additional chemicals which are hazardous and detrimental to human safety and the atmosphere could increase the reactivity and toxicity of the particles which may induce unintended adverse health effects due to their lack of consistency and compositional uncertainty [12, 13]. Green synthesis approaches are dramatically desirable provided their ability to reduce NP toxicity.

Nanotechnology is the field of science that investigates nano-range products, usually between 1 and 100 nm. This is a technology that operates at the nanoscale and brings the varied areas of science such as pharmaceuticals, dentistry, and bio-engineering [14, 15]. Green chemistry strategy is important for nanomaterials potential prospects. This field of nanoscience will ultimately result in the production of healthy, environmentally sustainable NPs, and be widely adopted in nanotechnology [16, 17]. The two significant methods for amalgamating NPs are “top-down” and “bottom-up” approaches using different methods such as crushing, sputtering, milling, thermal/laser ablation, etc. Suitable bulky content is broken down into small, fine particles in the top-to-bottom approach, while NPs are synthesized in the bottom-to-top strategy using biological and chemical methods through the self-assembly of atoms into new nuclei that develop into nanosize particles, whereas the bottom-up approach involves chemical reduction and electrochemical methods [18].

Metal oxides are of considerable concern to scientists, because of their improved surface composition and high surface area. Magnesium oxide (MgO) is one of the most important metal oxides with a wide range of applications in the catalysis [19–21] refractory materials [22], paint [23] and superconducting industries [24], biological, electrochemical, and medical fields [25]. Metal nanoparticles (NPs) have various applications in the biological, electrochemical, environmental [26–29], and medical fields [25] due to their specific properties such as large surface area to volume ratio, optical, magnetic [30], high surface energy compared to their bulk counterparts. Among different nanoparticles of metal oxide semiconductors (ZnO, SnO_2_, WO_3_, TiO_2_, etc.), magnesium oxide nanoparticles (MgO-NPs) earned considerable significance in the area of gas sensors due to their specific chemical and physical properties [31]. MgO is a major inorganic oxide and is considered a healthy substance for humans. MgO is used in the medical sector to treat different ailments as an antacid for heartburn and sore stomach [32]. In contrast to all other nanoparticles, magnesium oxide is very important because they have unique characteristics compared to bulk materials. Metal-based nanomaterials target several sites in living systems, and may also reduce the risk of growing drug resistance [16].

The excellent properties of MgO nanoparticles made them unique include high chemical stability, high photocatalytic activity, high electrical permittivity, non-toxicity, etc. Magnesium nanoparticles may have long-lasting antibacterial action due to its low volatility and high-temperature tolerant properties [33]. Based on the literature, synthesis of MgO-NPs with neem leaves [34], *Parthenium* [35], citrus lemon extract [36], *Brassica oleracea*, and *Punica granatum* peels [37] are available. Biological nanoparticles have been found safe, ecologically responsible, cost-effective, and ensures the complete removal of toxic chemicals [38]. Therefore, the green synthesis is still an unexplored area to achieve MgO-NPs, and it provides numerous research possibilities. Among the metal oxide nanoparticles, zinc oxide and magnesium oxide nanoparticles have obtained considerable focus, due to their unique biochemical and physicochemical and properties. Similarly, magnesium (Mg) is also a vital component for the development of the plant, acting as a powerhouse during the photosynthesis process. These nutrients, in comparison to the bulk, interact strongly with plants at the nano level by substantial absorption and accumulation. Thus, MgO-NPs has applications even in agriculture [39, 40].

Because of the abundance of effective phytochemicals in different plant extracts, plant biodiversity was commonly considered for the production of metal/metal oxide nanoparticles. Such constituents can reduce metal salts to metal nanoparticles [41]. The specific characteristics of these nanomaterials are explored for use in diagnostics, biomedical, antimicrobials, molecular signaling, catalysis, optical imaging, and biological device labeling [42]. The phytochemicals found in plant leaf extracts have an exceptional ability to minimize metal ions in a much shorter period compared to bacteria and fungi, allowing longer time for incubation. Thus, plant leaf extracts are considered the most suitable and beneficial route for both metal and metal oxide nanoparticle synthesis. Plant leaf extract plays a dual function by serving as both reduction and stabilization agents in the nanoparticle synthesis cycle to enable the synthesis of nanoparticles [43].

The plant leaf extract composition is also a vital aspect in the production of nanoparticles, for example, various plants produce differing amounts of phytochemicals. The major plant phytochemicals are flavones, terpenoids, carbohydrates, aldehydes, ketones, amides, and carboxylic acids, which are responsible for nanoparticle bio-reduction [44]. Flavonoids have different biological groups and are better able to suppress metal ions. According to tautomeric transitions in flavonoids, the reactive hydrogen atom is released from which enol-form is transformed into the keto shape. This cycle is achieved by converting metal ions to metal nanoparticles. In sweet basil (Ocimum basilicum) extracts, the main element in the synthesis of biogenic silver nanoparticles is the enol- to keto-transformation [45]. Plant extracts are made up of biomolecules of carbohydrates and proteins, which serve as a reduction agent to encourage the generation of metallic nanoparticles [10].

*Pterocarpus marsupium* Roxb is a large tree, grown widely on the eastern, western, and southern parts of India and Sri Lanka, from the Leguminosae family known as Vijaysar or Bijasar. Several parts of the *P*. *marsupium* tree (bark, heartwood, leaves, and flowers) have been used for medicinal purposes in Ayurveda for many years. Rajgovind [46] examined the usage of *Pterocarpus marsupium* heartwood for the production of metallic oxide nanoparticles and tested their efficacy against Gram-positive bacteria and Gram-negative bacteria. It has long been shown that heartwood is beneficial for diabetes [47], and is used to relieve inflammation. There has been reported antidiabetic, antihyperlipidemic, and antioxidant activity of flavonoids and phenolic content in the tree [48]. According to conventional reports, *Pterocarpus marsupium* heartwood is the potential source of drugs utilized as an anti-inflammatory, astringent, antihelmintic, diabetes, leprosy, skin disease, diarrhea, bronchitis, asthma, and hair grayness.

The most important bioactive components are terpenoids, saponins, flavonoids, tannins, phenolic compounds, and alkaloids. Recently, furthermore, three major phenolic constituents isolated from the *P*. *marsupium* heartwood are marsupin, ptrerosupin, and pterostilbene [49]. Marsupin and pterostilbene substantially reduced hyperglycemic rat blood glucose levels [50]. Rastogi and Mehrotra [51] isolated 6-6 glucoside tetrahydroxyisoflavone 5,7,2-4, which is a powerful antioxidant thought to prevent heart diseases. So many of these isolated compounds can serve as a potential supply of natural antioxidants from the different parts of the extract of *P*. *marsupium*. The plant’s heartwood juice is considered to contain polyphenolic compounds (such as diphenyl propane flavonoids, sesquiterpenes, and derivatives), which exhibit high antioxidant, anti-inflammatory, antidiabetic, antimicrobial, and anticancer activity, and is used to treat diabetes, jaundice, ulcer, gastritis, and so on [52]. This plant was chosen for its high content of flavonoids and phenolics. Flavonoids have been described as crucial for the green synthesis of nanoparticles [53].

In the context of the above discussion, the current research investigates the synthesis of MgO-NPs by using *P*. *marsupium* heartwood extract and their biomedical applications. This is the first research to determine the impact of green synthesized MgO-NPs on antidiabetic and anti-inflammatory behaviors, to the best of our knowledge. This study thus offers an efficient, inexpensive, benign means for the production of metal nanomaterials with their therapeutic applications.

## Methods

### Collection of plant

The plant *Pterocarpus marsupium* heartwood was collected from Tirupati area and was identified and authenticated by Dr. K. Madhava Chetty, Assistant Professor, Department of Botany, Sri Venkateswara University, Tirupati (Voucher no. 1587 has been deposited in a herbarium). The field studies were conducted in accordance to the local legislations and have taken necessary permissions. The plant was subsequently washed, dried in shade, subjected to fine grinding, and placed in a dry place in an airtight container.

### Preparation of *P*. *marsupium* heartwood aqueous extract

For 30 min, 50 g of coarse powder was boiled in 500 mL of distilled water. Then aqueous extract was cooled, filtered with a Whatman No. 1 filter paper, and refrigerated for further use.

### Total phenolic content

The total phenolic content (TPC) was estimated spectrophotometrically with the Folin-Ciocalteu reagent using gallic acid as standard [54]. *P*. *marsupium* aqueous heartwood extract (2 mL) was combined with a diluted reagent of 10 mL Folin-Ciocalteu (1/10 with distilled water). Eight milliliters of sodium carbonate was further added to the solution after incubation for 2 min. The process solutions were then kept for 2 h with occasional shaking at 37 °C in the dark. Estimated absorbance to be 765 nm, at which a standard curve was made by taking gallic acid, at a concentration range of 50 to 500 μg/mL. The findings are calculated in μg of an equivalent gallic acid (GAE) per mL of *P*. *marsupium* extract. All tests are carried out in triplicate

### Total flavonoid content

The aluminum chloride colorimetric method was used for the calculation of flavonoid content in *P*. *marsupium* aqueous extract [55]. One milliliter of *P*. *marsupium* heartwood extract (50 mg/mL) was mixed with 4 mL of distilled water and 0.3% sodium nitrate was mixed. The reaction solution was subsequently incubated for 10 min and 0.3% of aluminum chloride was added and the solution was maintained for 6 min. After which, 1 mol/L of 2 mL sodium hydroxide solution was mixed, adding up to 10 mL of the final amount with the distilled water to the reaction mixture. The mixture remained in position for another 15 min, and the absorption was estimated at 415 nm. The total flavonoid content (TFC) was calculated from the calibration curve of quercetin (10–50 μg/mL) as standard, and the result was expressed as mg quercetin equivalent (QE) per g of dry extract weight.

### Biosynthesis of MgO nanoparticles

The magnesium oxide nanoparticles are synthesized according to Gaurav. S [56] with slight modifications. Thirty milliliters of *P*. *marsupium* extract prepared has been added dropwise to a 150 mL of freshly prepared magnesium nitrate solution and 1 M NaOH was also added dropwise at 80 °C for 6 h under continuous stirring using a magnetic stirrer. The modification of color from colorless to brown observed with the addition of magnesium nitrate solution determines the production of nanoparticles. The prepared mixture was then centrifuged at 12,000 rpm for 30 min and the collected pellet was washed several times with ethanol to eliminate any impurities and was calcinated at 400 °C in a furnace to extract dried MgO-NPs.

### Characterization of MgO-NPs

Biosynthesized MgO nanoparticles and *P*. *marsupium* heartwood extract and plant extract are characterized by the UV-visible spectroscopy (Shimadzu, Tokyo, Japan). One milliliter of *Pterocarpus marsupium* wood extract was taken in a 10 mL flask and diluted with purified water and phosphate buffer at pH 7.4 was taken as blank and UV-visible spectra are obtained in the range of 200–800 nm. The surface morphological characteristics such as shape, size, and composition of MgO-NPs are monitored by scanning electron microscopy–energy-dispersive X-ray spectroscopy (SEM-EDX) using Zeiss SEM machine with spectral imaging system and transmission electron microscopy (TEM) using FEI-Tecnai G2 20 Twin, VIT university. The test sample (MgO-NPs) for SEM investigation was developed by placing the filtered lyophilized nanoparticles on the network, which was allowed to dry under a mercury light after 10 min of drying for SEM study. Test for TEM investigation was developed by placing a small drop of suspended nanoparticles on a carbon-covered copper network and enabling the dissipation of water into a vacuum dryer. TEM images were scanned on the grid containing MgO-NPs. MgO-NPs sample analysis by X-ray diffraction (XRD) was performed on a Miniflex 600 Powder XRD instrument, Osmania University operating at 40 kV with a current of 30 mA using Cu Ka at a scanning range of 10–80. Fourier transform infrared spectroscopy was carried out to examine the formation of magnesium oxide nanoparticles mediated by the functional groups in *P*. *marsupium*. The spectra of the considerable number of test samples (MgO-NPs, PE) was reported by KBr pellet production at room temperature spectrometrically (Shimadzu FT-IR spectrophotometer). The range was preserved at a resolution of 4 cm^−1^, between 4000 and 400 cm^−1^. FTIR analysis was conducted to classify the biomolecules essential for capping and stabilizing synthesized metal nanoparticles. Zeta potential, particle size, and particle size distribution of magnesium oxide nanoparticles were measured using a particle size analyzer based on laser light scattering (Zetasizer NS 3000, Malvern Instruments). To avoid aggregation, the freshly made solution containing MgO-NPs is dispersed in distilled water was ultra-sonicated at 90% amplitude of probe for 10 min. MgO-NPs are dispersed in water at a concentration of 40 μg/mL, and mixing was performed by probe sonication for 10 min just before estimation. The lyophilized form of MgO-NPs is dispersed in water to achieve the appropriate spreading intensity of nanoparticles. Malvern zeta-size analyzer has calculated the particle size. The zeta potential was calculated using Zeta Sizer (Malvern Instruments) with polycarbonate cells with gold-plated electrodes, and utilizing water as a source for sample preparation. Zeta potential defines the surface potential of magnesium oxide nanoparticles, which is important for characterizing nanoparticle stability. The study was performed in triplicate fashion, and average values were reported with standard deviation.

### Minimum inhibitory concentration

The test microorganisms include Gram-positive bacteria *Staphylococcus aureus* (MTCC 3160), Gram-negative bacteria *Escherichia coli* (MTCC 1683). According to Shaghufta Perveen [57], minimum inhibitory concentration (MIC) of plant extract, and magnesium oxide nanoparticles synthesized with *Pterocarpus marsupium* were determined using the broth dilution process. Sterilized nutrient broth (5 mL) was taken into each test tube for minimum inhibitory concentration assay and inoculated with 100 μL of the freshly made test strain. Next, various concentrations of *Pterocarpus marsupium* (PM), and biogenic MgO-NPs (5, 10, 15, 20, 25, 30, 35 μL) are added in the test tubes and incubated under an orbital shaker with 120 rpm for 24 h at 37 °C. After 24 h, each test tube was evaluated for turbidity at 600 nm using a Spectrophotometer. The bacterial colonies were concurrently treated with MgO-NPs, control (i.e., sterile media devoid of test solution), and blank (i.e., sterile media devoid of inoculums). MIC has been described to be the lowest MgO-NPs concentration inhibiting bacterial growth. All the assays were conducted in triplicates.

### Antioxidant activity

MgO-NPs with *P*. *marsupium* extract were tested for their ability to neutralize radical PPP (2,2-diphenyl-1-picrylhydrazyl), which was demonstrated by a reduction in DPPH methanol solution absorbed in the reaction [58]. The testing methods are focused on the analysis of anti-oxidation in MgO-NPs, which are synthesized with *Pterocarpus marsupium* extract. Different dilutions are prepared between 10 and 320 μg/mL. To calculate the changes in the absorption rate, a spectrophotometer was used. Each test tube was kept 30 min away from light. The dilutions were combined with 1.0 mL of DPPH reagent at 0.1 mM concentration in methanol. The DPPH reagent was set up 24 h in advance. The absorption was examined at a wavelength of 515 nm followed by 30 min of shaking. Next, taken 1 mL of water and methanol in each test tube and were used as reference. The absorption of the DPPH solution was calculated before measuring sample absorbance. The sample represented free DPPH radicals scavenging activity as a percentage of free radical inhibition and was measured using the following formula:

$$ \%\mathrm{inhibition}=\frac{A_O-{A}_t}{A_T}\times 100 $$

where *A*_0_ was the control absorbance and where *A*_t_ was the absorbance in the sample presence. All experiments were run in triplicate, and mean values were plotted on the graph.

### Alpha-amylase inhibition activity

Alpha-amylase is an enzyme which helps to split large, insoluble molecules of starch into soluble molecules found in pancreatic juice and saliva. Different concentrations of prepared MgO-NPs were taken in various testing tubes from 1 to 5 mg and the volume was developed up to 0.5 mL with a phosphate buffer of pH 6.9. By using 1 mL of phosphate buffer, a blank was measured. Then 0.5 mL of α-amylase (0.5 mg/mL) was added to the solution. Thereafter, the solution was incubated for 10 min at 25 °C. Subsequently mixed, 0.5 mL of 1% starch solution into 0.02 M sodium phosphate buffer of pH 6.9 to all test tubes and incubated for 10 min at 25 °C. The reaction was halted by introducing 1.0 mL DNS, and the reaction mixture was kept in a boiling water bath for 5 min and cooled to room temperature. The solution was made with distilled water up to 8 mL, and the absorbance was read in the spectrophotometer at 540 nm against a blank solution [59]. The percentage inhibition of α-amylase enzyme was calculated using the formula:

$$ \mathrm{Inhibition}\ \left(\%\right)=100\times \left[\mathrm{Control}-\mathrm{Test}/\mathrm{Control}\right] $$

### Inhibition of albumin denaturation

The reaction mixture (5 mL) consisting of 0.2 mL of egg albumin (fresh hen’s egg), 2.8 mL of phosphate-buffered saline (pH 6.4), and 2 mL of varying extract concentrations to exceed 20, 40, 60, 80, 100 μg/mL of final concentrations. Similar volume of double distilled water was served as control. The mixtures were then incubated in a Biological oxygen demand incubator at (37 ± 2 °C) for 15 min, and then heated for 5 min at 70 °C. Their absorption was estimated at 660 nm after cooling using the blank. Dicflonec sodium was used as a standard drug at final concentrations and tested equally for absorbance calculation [60]. The percentage inhibition of albumin denaturation was calculated by using the formula:

$$ \%\mathrm{inhibition}=100\times \Big(\left[{V}_t/{V}_c\right]-1 $$

where *V*_t_ = absorbance of the test sample and *V*_c_ = absorbance of control

## Results

### Total phenolic content

The total phenolic content of *P*. *marsupium* heartwood extract was calculated spectrophotometrically using Folin-Ciocalteu reagent and was expressed as GAE/mL of plant extract. The standard gallic acid curve was used to estimate the actual quantity of phenolic compounds in heartwood extract. Phenolic content in the test sample was found to be 89.76 ± 1.7 GAE per mL.

### Total flavonoid content

The complete flavonoid content of *P*. *marsupium* aqueous extract was evaluated using an aluminum chloride procedure. The standard quercetin curve was used to calculate the actual quantity of flavonoid compounds in heartwood extract. Flavonoid content in the test sample was found to be 54.34 ± 1.2 μg QE.

### Physical appearance and UV spectroscopy analysis of phytoassisted MgO-NPs

**Fig. 1:**
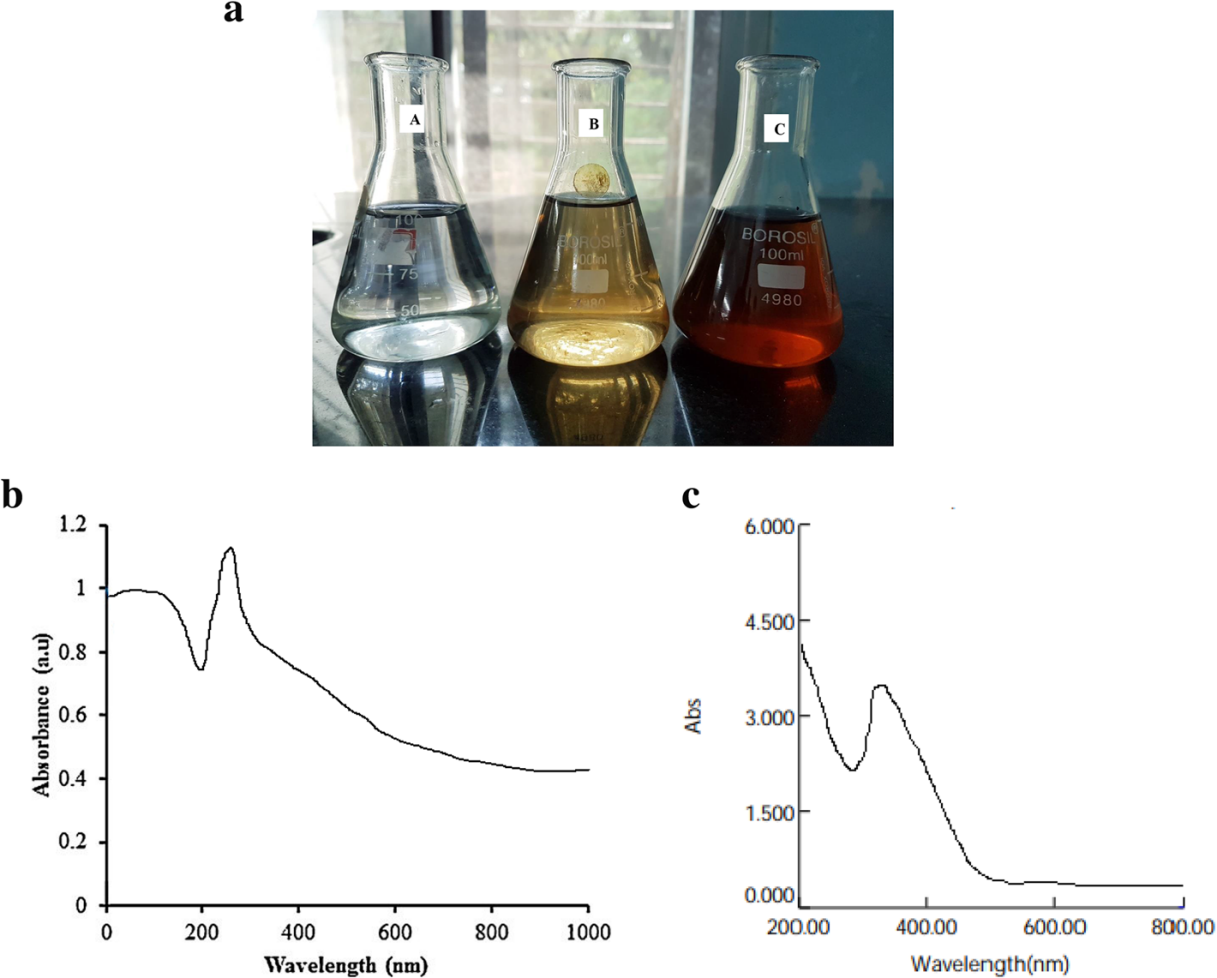
**a** A: magnesium nitrate solution, B: before the synthesis of nanoparticles, C: after synthesis of nanoparticles. **b** UV-Visible spectrophotometric analysis of biosynthesized magnesium oxide nanoparticles. **c** UV-Visible spectrophotometric analysis of *Pterocarpus marsupium* heartwood extract

During the formation of magnesium oxide nanoparticles synthesized from aqueous heartwood extract of *Pterocarpus marsupium*, the colorless magnesium nitrate solution changed to dark brown as shown in Fig. 1a. The prepared phytosynthesized magnesium oxide nanoparticles and *P*. *marsupium* extract were confirmed by UV-Vis spectroscopy. At room temperature, for 24 h the prepared nanoparticle solution was kept aside. The UV-visible MgO-NPs synthesized with *P*. *marsupium* heartwood extract after 24 h showed in Fig. 1b. The absorption peak at 310 nm indicates that MgO-NPs are synthesized. Therefore, it was evident that Mg(NO_3_)_2_ was reduced to MgO. The UV-Vis spectrum of plant extract shows a wavelength at 281 nm (Fig. 1c).

### XRD analysis

**Fig. 2:**
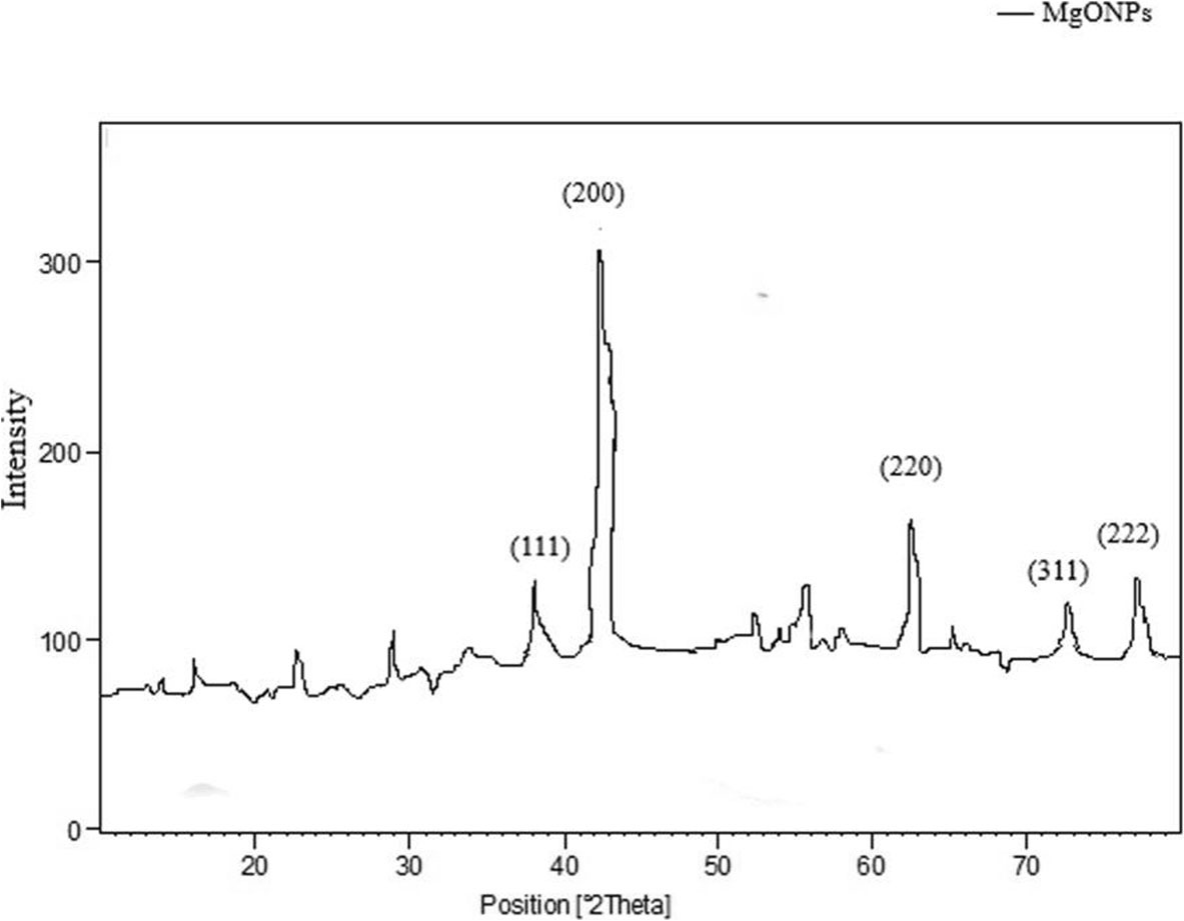
XRD pattern of green synthesized MgO nanoparticles using *P*. *marsupium* heartwood extract

Figure 2 shows the XRD pattern of magnesium oxide nanoparticles obtained with *Pterocarpus marsupium* heartwood extract. After the reaction, the intense diffraction peaks at 2θ values of 37.76°, 43.12°, 64.97°, 74.45°, and 78.12° assigned to the (111), (220), (220), (311), and (222) planes of a faced center cubic (FCC) lattice of magnesium respectively and suggest that the magnesium oxide nanoparticles are crystalline [61]. The patterns comply well with standard diffraction data of magnesium oxide nanoparticles (JCPDS file No. 89-4248). On the diffraction peaks with the Debye-Scherrer equation below, the average crystallite dimension was calculated. In particular, there are only a few other peaks in the spectrum which indicates the lack of metallic impurities.

$$ D= k\lambda \left(\beta\ \cos \theta \right) $$

where *D* is the particle size of the crystal, *k* is the Sherrer constant (0.9), λ is the X-ray wavelength (0.15406 nm), β is the width of the XRD peak at half-height, and θ is the Bragg diffraction angle.

### FT-IR spectroscopic analysis

**Fig. 3:**
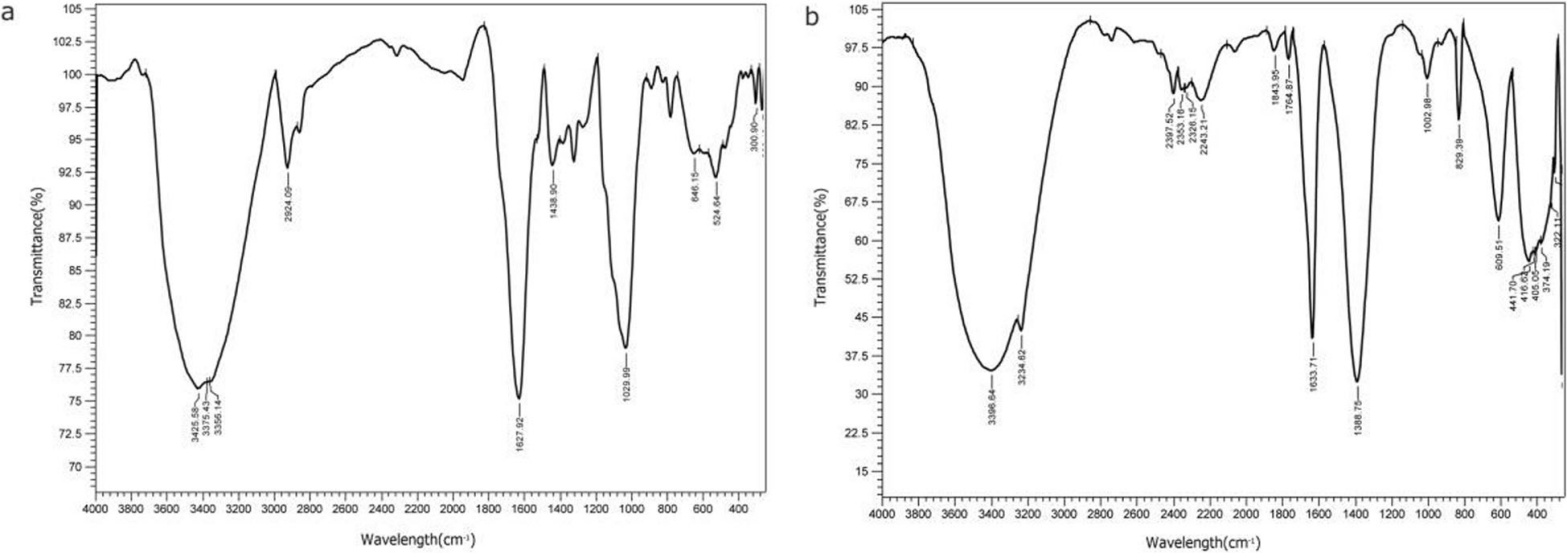
**a** FTIR image of *Pterocarpus marsupium* heartwood aqueous extract. **b** FTIR image of *Pterocarpus marsupium* synthesized MgO nanoparticles

Fourier transform infrared spectroscopy (FT-IR) studies of magnesium oxide nanoparticles synthesized from *Pterocarpus marsupium* extract and plant extract were performed to characterize the chemical nature of the nanoparticles as shown in Fig. 3. The study shown sharp absorption peaks at 3400 to 3300, 3356, 645, and 524, 1625 cm^−1^ for plant extract (Fig. 3a) and intense peaks of MgO-NPs are shown at 3400 to 3300, 3396, 2924, 1029, 1625,1633, 405, 416, and 441 cm^−1^ (Fig. 3b). The FT-IR response proves the existence of alkaloids, phenolic compounds, amino acids, flavonoids in the *Pterocarpus marsupium* heartwood extract.

### Zeta potential, particle size, and PDI of MgO-NPs

**Fig. 4:**
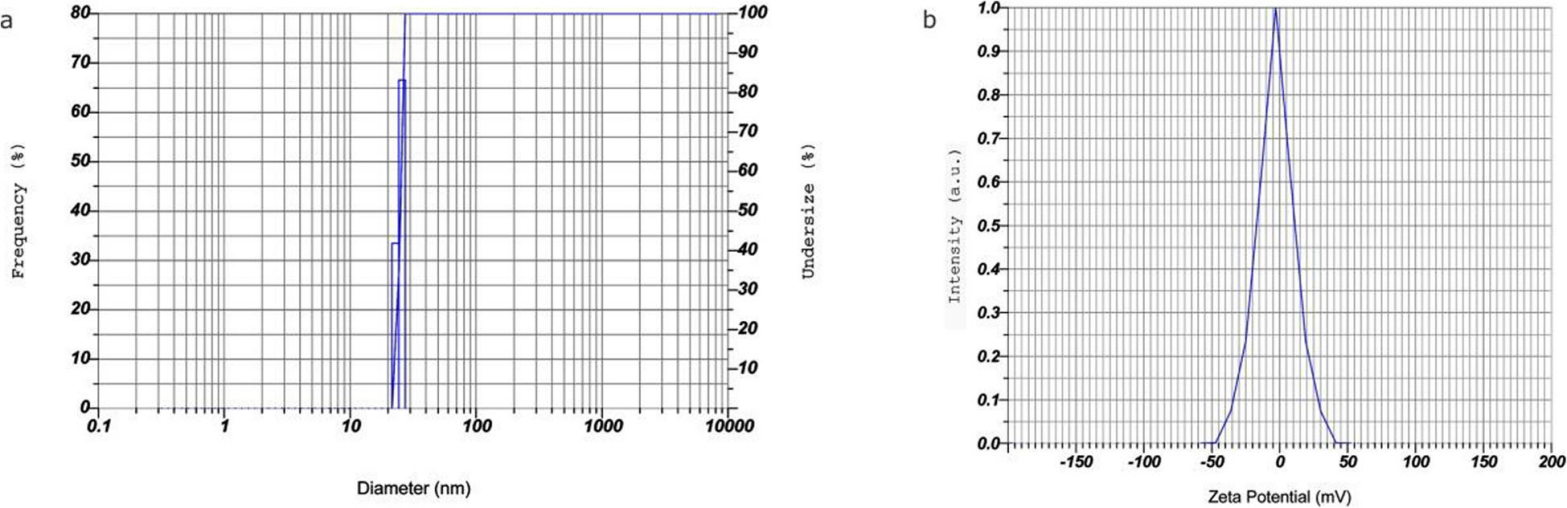
**a** Particle size of the green synthesized MgO-NPs using *P*. *marsupium* heartwood extract. **b** Zeta potential of green synthesized MgO-NPs using *P*. *marsupium* heartwood extract

Using a dynamic light scattering technique, the mean particle size, polydispersity index (PDI), and zeta potential of MgO nanoparticles synthesized using *Pterocarpus marsupium* were studied. The surface charge of biosynthesized MgO nanoparticle was measured at zeta potential of − 2.9 mV using dynamic light scattering (DLS) was graphically represented (Fig. 4b). The average size of MgO-NPs synthesized from *P*. *marsupium* is 25 nm (Fig. 4a). The PDI value of MgO-NPs synthesized from *Pterocarpus marsupium* is 0.248.

### SEM studies of MgO-NPs

**Fig. 5:**
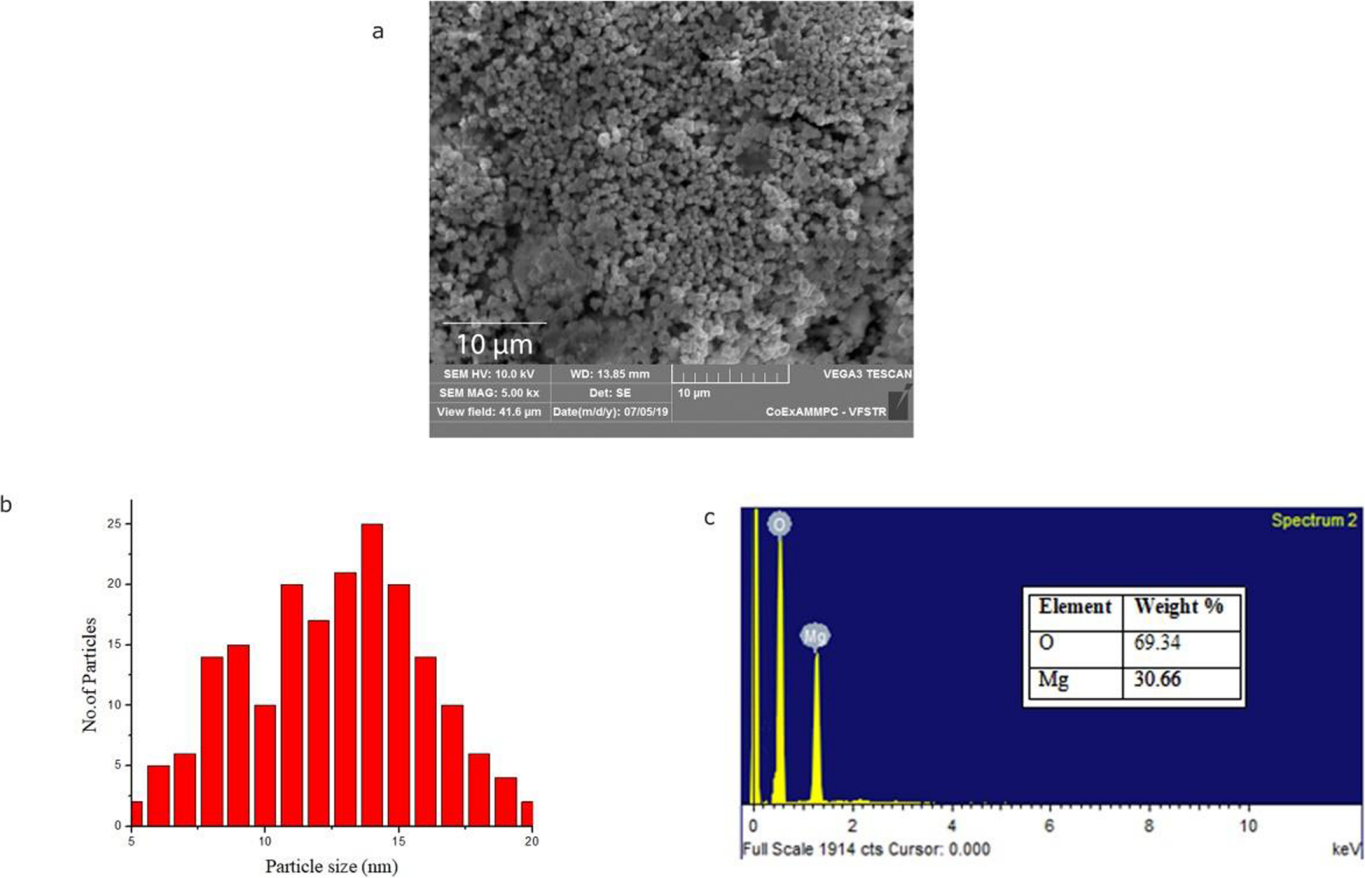
**a** SEM images of biosynthesized MgO nanoparticles at scale bar 10 nm. **b** SEM images of biosynthesized MgO nanoparticles at scale bar 10 nm. **c** EDS profile of biosynthesized MgO nanoparticles

Once the size of the nanoparticle was confirmed, it was subjected to electron microscopy for detailed morphological studies. The scale at 10 μm (Fig. 5a) of magnesium oxide nanoparticles synthesized using *P*. *marsupium* heartwood extract along with energy-dispersive X-ray spectroscopy (EDS) profiles are shown in Fig. 5c, which indicates the MgO-NPs presence, with peaks at between 0.5 and 1.5 kV. To investigate the average size of the nanoparticles, the histogram analysis was performed (Fig. 5b). In contrast, the MgO-NPs are spherical, well scattered showing a large surface area to volume ratio, integrated, and bigger clusters are formed.

### TEM analysis of MgO-NPs

**Fig. 6:**
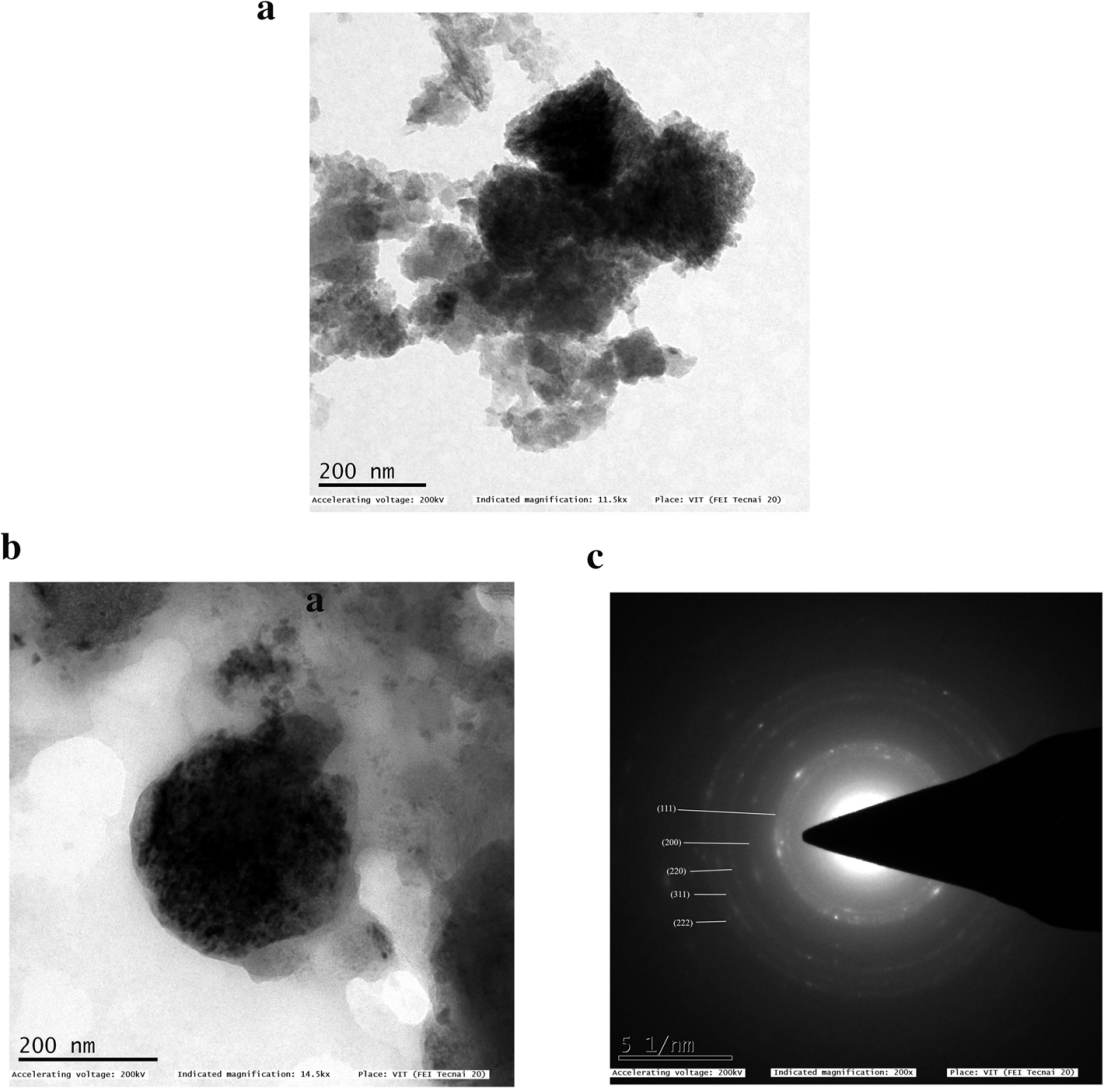
**a** TEM micrograph of MgO nanoparticles synthesized with *Pterocarpus marsupium* heartwood extract at scale bar 200 nm. **b** TEM micrograph of single MgO nanoparticle at scale bar 200 nm. **c** SAED pattern of single magnesium oxide nanoparticle

The TEM images of biosynthesized MgO nanoparticles have been shown (Fig. 6). In the low magnification picture at a scale bar of 200 nm of single and cluster of MgO nanoparticles (Fig. 6a, b), it can be seen that all the particles were random in size and exhibit mono-dispersity, whereas under a highly magnified TEM micrograph at a bar of 200 nm (Fig. 6a), a reasonably good understanding of shape and size can be obtained; the size of the nanoparticle is between 10 and 20 nm and spherical shaped. Figure 6c displays selective area electron diffraction (SAED) patterns of a single MgO nanoparticle, where the white spot array supports the nanoparticle’s crystalline nature.

### Biomedical applications of *Pterocarpus marsupium* synthesized MgO-NPs antioxidant activity by DPPH radical scavenging

**Fig. 7:**
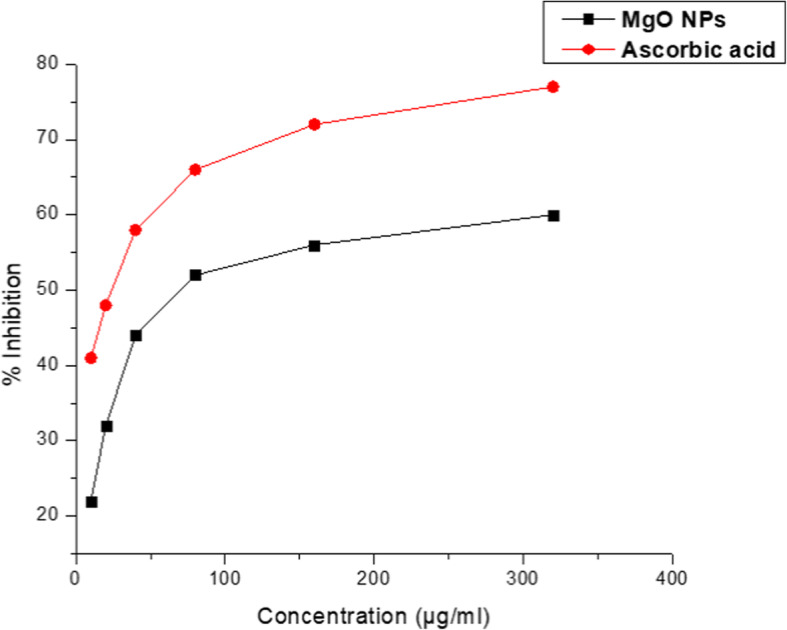
DPPH radical scavenging activity of MgO nanoparticles synthesized with *Pterocarpus marsupium* heartwood aqueous extract

MgO-NPs synthesized with *P*. *marsupium* heartwood extract was determined to demonstrate antioxidant activity, with a radical DPPH sample (Fig. 7). The MgO-NPs scavenged free radicals dependent on concentration showed percentage inhibition at a minimum range of 10 μg/mL at 22.34 ± 1.65 with a maximum range of 320 μg/mL at 52.43 ± 0.54 respectively. This study was carried out by measuring the IC_50_ parameter for the DPPH scavenging activity of MgO-NPs synthesized with *P*. *marsupium* heartwood extract as shown in Table 1. This factor decides how free radicals can be scavenged. Increasing the IC_50_, the antioxidant is more reactive.

**Table 1:** In vitro antioxidant activity of MgO-NPs synthesized from *Pterocarpus marsupium* heartwood extract by DPPH radical scavenging

Sample	IC_50_ (μg/mL)
Ascorbic acid	38.73
MgO-NPs	89.67

### Minimum inhibitory concentration

**Fig. 8:**
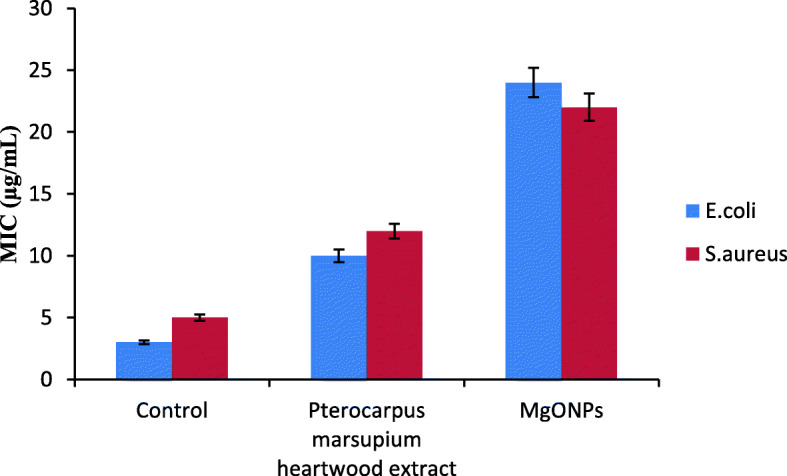
Graphical representation showing minimum inhibitory concentrations of MgO-NPs synthesized from *P*. *marsupium* heartwood extract against *E*. *coli* and *S*. *aureus*

MIC of developed MgO-NPs, PM, and control against Gram-positive and Gram-negative are shown in Table 2 and Fig. 8. The MIC values of MgO-NPs against both bacteria were found to be between 10 and 25 μg/mL.

**Table 2:** Minimum Inhibitory Concentration values of MgO-NPs synthesized from *P*. *marsupium* heartwood extract against *E*. *coli* and *S*. *aureus*

	Control	*Pterocarpus marsupium* Extract	Biogenic MgO-NPs
*E. coli*			
MIC (μg/mL)	3 ± 0.139	10 ± 0.261	24 ± 0.439
*S. aureus*			
MIC (μg/mL)	5 ± 0.121	12 ± 0.070	22 ± 0.168

Data represents the average of three experimental values ± SD

### Alpha-amylase inhibitory activity of MgO-NPs

**Fig. 9:**
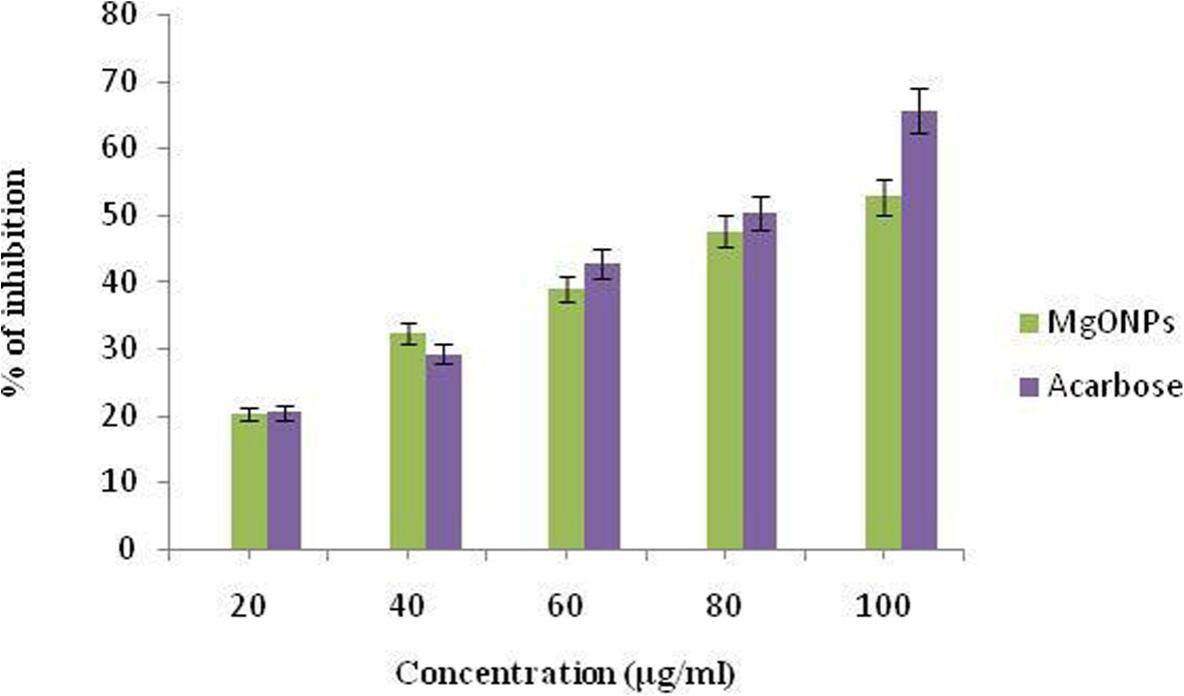
Representing the percentage inhibition of alpha-amylase by biosynthesized MgO-NPs and Acarbose at different concentrations

Figure 9 shows the effectiveness of MgO-NPs synthesized with *P*. *marsupium* in inhibiting α-amylase. The percentage inhibition of α-amylase by the MgO-NPs was investigated within a concentration range of 20–100 μg/mL, and 56.32 was found to be the IC_50_ value as shown in Table 3.

**Table 3:** Alpha-amylase inhibitory activity by *Pterocarpus marsupium* synthesized MgO-NPs

Treatment	Concentration (μg/mL)	Percentage of inhibition (%)	IC_50_
MgO-NPs synthesized with *Pterocarpus marsupium*	20	20.23 ± 1.11	56.32
	40	32.21 ± 1.45	
	60	38.87 ± 1.31	
	80	47.56 ± 1.45	
	100	52.72 ± 1.52	
Acarbose	20	20.45 ± 1.01	228.67
	40	29.23 ± 1.54	
	60	42.65 ± 1.56	
	80	50.37 ± 1.87	
	100	65.56 ± 2.03	

Values are expressed in terms of mean ± SEM (*n* = 6)

### Effect on protein inhibition

Denaturation of proteins is a common process of inflammation. In diabetes, the stimulation of cytokines activates the membrane inflammatory responses and tissue proteins. Protein denaturation levels are a measure of the severity of the inflammation. Results indicate that the MgO-NPs help inhibit the process of protein denaturation which therefore contributes to a decrease in the process of inflammation in patients with diabetes.

**Fig. 10:**
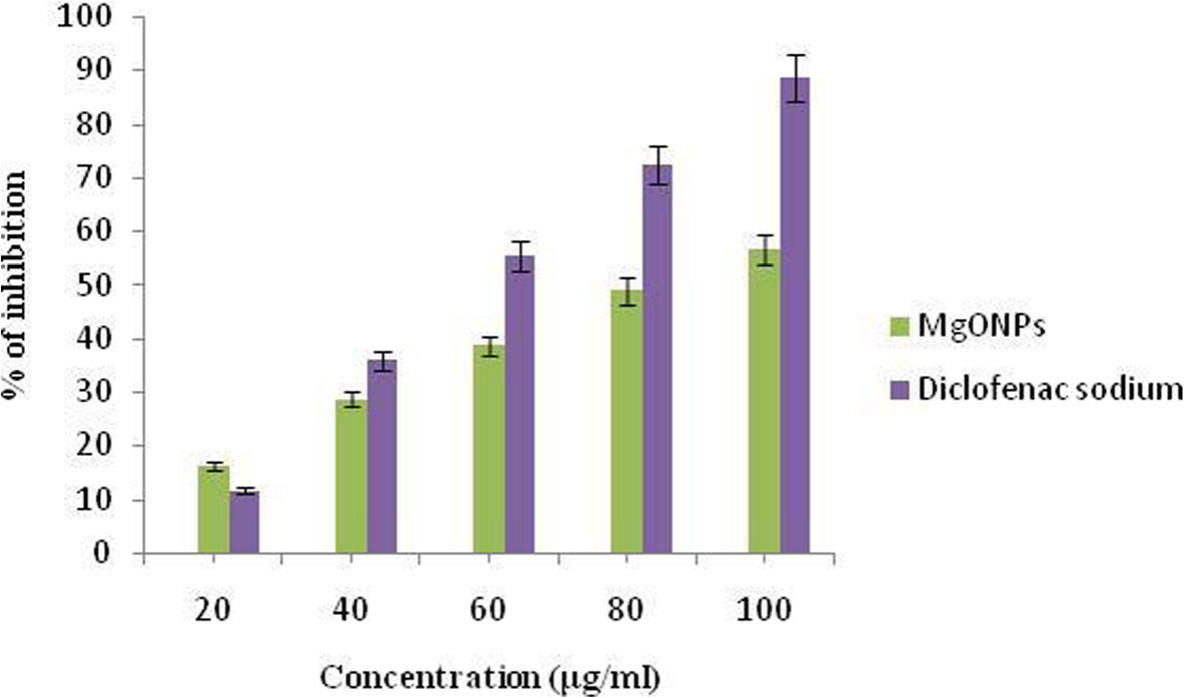
Representing the albumin inhibitory activity calculated at different concentrations of biosynthesized MgO-NPs and Diclofenac sodium

Figure 10 demonstrates the in-vitro anti-inflammatory activity of MgO-NPs on inhibiting protein denaturation. The minimum inhibition observed by the synthesized nanoparticles at 20 μg/mL concentration is 16.26 ± 1.01%. As shown in Table 4, the IC_50_ was found to be 81.69.

**Table 4:** Albumin denaturation activity by *Pterocarpus marsupium* synthesized MgO-NPs

Treatment	Concentration (μg/mL)	Percentage of inhibition (%)	IC_50_
MgO-NPs synthesized with *Pterocarpus marsupium*	20	16.26 ± 1.01	81.69
	40	28.64 ± 1.23	
	60	38.58 ± 1.27	
	80	48.92 ± 1.54	
	100	56.53 ± 1.31	
Diclofenac sodium	20	11.63 ± 1.02	154.78
	40	35.89 ± 1.37	
	60	55.45 ± 1.43	
	80	72.38 ± 1.54	
	100	88.48 ± 2.03	

Values are expressed in terms of mean ± SEM (*n* = 6)

## Discussion

### Characterization of MgO-NPs

#### Physical appearance and UV spectroscopy analysis of phytoassisted MgO-NPs

A drop in the color intensity of the extract was observed as an initial confirmation of the creation of the MgO-NPs. This is due to the fact that when undertaking the reduction and stabilization processes for the synthesis of nanoparticles, the phytochemicals of the plant extract were degraded [62]. The presence of a characteristic absorption band associated with MgO-NPs at 310 nm further supports the development of nanoparticles by the green method used, apart from the color transformation of the extract (from colorless to brown) (Fig. 1a). A single peak appearance in the UV-spectrum of MgO-NPs shows that the prepared NPs is iso-morphological [63].

The color modification is due to the phyto-reduction by the *P*. *marsupium* aqueous extract of the magnesium nitrate and is apparent for the successful biosynthesis of nanoparticles.

Phytochemical analysis proved the presence of glycosides, carbohydrates, flavonoids, tannins, saponins, phenolic compounds, and alkaloids in various parts of the plant. It is also a rich source of biomolecules like Epicatechin, marsupin, ptrosupin, pterostilbene, and 5,7,2-4 tetrahydroxy isoflavone 6-6 glucoside [38]. The result of UV–Vis indicates that the reductive biomolecules in the heartwood extract were able to perform the task of bio-reduction resulting in the formation of MgO-NPs. As shown in the Fig. 1b, the absorption peak lies in the range of 200–800 nm. In this process, the lack of sharp peaks indicates the production of nanoparticles at different sizes and supports the findings of the visible-ultraviolet spectrum and electron microscopy.

### XRD analysis

The average crystalline size of the MgO-NPs produced from the values was determined to be 16.76 nm. Individual crystallization of the capping agents was excluded out as part of the purification procedure due to the process of centrifugation and particle dispersion in water following the formation of nanoparticles. Also, the expansion of peaks in the stable XRD pattern is due to the influence of particle size.

### FT-IR spectroscopic analysis

Because of the presence of alcoholic or phenolic groups the large peak can be seen in the area between 3400 and 3300 cm^−1^ in Fig. 3a and b. The infra-red band at 3356 cm^−1^ was shown in Fig. 3a for the O-H bond vibrations of a hydroxy group, a slight decrease in peak intensity, and shift of the peak from 3356 cm^−1^ (Fig. 3b) to 3396 cm^−1^ in Fig. 3a indicates the role of organic molecules in the development of MgO nanoparticles. This broad peak at 3396 cm^−1^ helps to stabilize the particles crystal growth, while restricting the particles size and preventing them from agglomeration (Fig. 3b). The peak at 2924 cm^−1^ suggests stretching of alkynes (Fig. 3b). The strong band at 1029 was allocated to the C-O stretching vibrations of alcohol. Additional peaks at 645 and 524 can be assigned to bending modes of aromatic compounds (Fig. 3a). The band at 1408 is assigned to the methylene scissoring vibrations from the proteins. The most extreme peak at 1625 cm^−1^ and 1633 cm^−1^ indicates C=O, typical of the flavonoid structure found in the aqueous extract of *Pterocarpus marsupium* as shown in both Fig 3a and b. The absorption peak at 1388 cm^−1^ was subjected to C-H bending vibrations of an aromatic tertiary amine group (Fig. 3a).

The peaks observed at 405, 416, and 441 cm^−1^ indicates the formation of MgO-NPs (Fig. 3b). Such peaks are observed due to vibrations of magnesium oxide. Likewise, MgO nanoparticles synthesized by Tamilselvi [64] generated large FT-IR transmission peaks at 449, 511, 584, and 671 cm^−1^, due to the presence of 103–105 magnesium oxide vibrations. The variations and changes in MgO vibrations might be caused by interactions with functional groups of plant extracts or by the use of different precursors. The aliphatic compounds (Alkynes and alkanes) found in *Pterocarpus marsupium* is converted into aromatic amine groups because the magnesium precursor has nitrate in the solution for preparing MgO nanoparticles. This transition may be linked to variations, such as the oxidation, reduction, or degradation of phytochemical compounds during nanoparticle development [11]. The existence of bioactive compounds in PM was confirmed by FT-IR spectra. These bioactive compounds were assumed as reducing and capping contributors for MgO-NPs [65].

### Zeta potential, particle size, and PDI of MgO-NPs

The existence of negatively charged oxide on the surface of the magnesium oxide particle indicates a negative zeta potentiality. Since magnesium oxide has oxygen on its surface that specifies its surface charge, it oxidizes the phytochemicals at their oxidation rate, where oxygen is crucial to the formation of magnesium oxide that defines particle stability [63]. All the particles vary in average sizes between 15 and 25 nm. The degree of “non-uniformity” when particles are distributed in a solution is defined as polydispersity. The polydispersity index is a measure of a sample’s dispersity in solution. A fully uniform sample will have a PDI of 0. PDI in the 0.2–0.6 range is appropriate because particle distribution is slightly polydisperse [66].

### SEM studies of MgO-NPs

Aggregation of particles is may be due to the Van der Waal forces and interactions between the magnesium oxide nanoparticles [67]. All of nanoparticles are in the range between 5 and 20 nm (Fig. 6b). The average size of the MgO-NPs was calculated to be 11.68 nm. The surface morphology presented has beneficial applications in catalysis [68] and medicine [69].

The signal properties of magnesium and oxygen were further investigated by the EDS profile. The EDS profile is supporting evidence of pure magnesium oxide nanoparticles. It shows the weight of magnesium at 30.66% and of the weight of oxygen at 69.34%. Graph shows the standard EDS profile of MgO. From the EDS graph, it is verified that the synthesized NPs are composed of only Mg and oxygen. We have not detected any other peaks, which again suggest that the synthesized NPs are pure MgO-NPs.

### TEM analysis of MgO-NPs

Interestingly, TEM images show that most of the particles are not in direct contact but are divided by a relatively standardized inter-particle space. The presence of lines also indicates the crystallinity of MgO nanoparticles which was otherwise deficient in non-metallic organic compounds and molecules. It is also evident from the pictures that the materials are agglomerated attributed to low stability. Particulate agglomeration can be minimized by adjusting the sample’s pH to increase the electronegativity charge [70]. The TEM finding also follows SEM morphology (Fig. 5), which has demonstrated closer-size uniformity of particles. The calculation of the average particle size of the MgO-NPs from the SEM micrograph (11.68 nm) aided by ImageJ contrasts with that reported from the TEM micrograph (13.28 nm), thereby validating the size parameter of the MgO-NPs. The average size of MgO-NPs synthesized using *Chloromolaena odorata* leaf extract was found to be 12.3 nm, which is almost close to the size of NPs obtained in the current research [71].

Acquired TEM results can be compared with the results obtained by the synthesis of SAED pattern of single MgO-NP displayed in Fig. 6c shows concentric circles with intermittent bright spots corresponding to crystalline ring pattern for a face-centered cubic structure, suggesting the strong crystalline nature of the particles. SAED pattern with spots and spongy pattern also provides support for its polydisperse existence as well. The rings can be attributed to the diffraction from the MgO planes (111), (200), (220), (311), and (222) corresponding to the crystalline nature of nanoparticles. As verified by the XRD study, these TEM diffraction rings may be assigned for their respective MgO lattice planes. The properties showcased by the magnesium oxide nanoparticles might be the outcome of the effective capping capacity of the bioactive compounds present in the plant extract along with the UV irradiation passing through the test sample in ethanol medium, which enhanced particle dispersion and homogeneity [67].

### Antioxidant activity by DPPH radical scavenging

This approach lowers the radical stable nitrogen 2, 2-diphenyl-1-picrylhydrazyl (DPPH) present in the measured sample, resulting in a decrease in absorption measurably at 515 nm. The oxygen-donating substances form the reduced DPPH, the solution thus loses its violet color. In this study, the effective radical solution is violet which shows that the earlier unknown electron is paired. The color change of the DPPH solution indicates its free radical scavenging property after application by the antioxidant solution. Standard ascorbic acid was taken as a control for the study. The plants are rich in antioxidants with various phytochemicals involved in antioxidant activity by neutralizing reactive oxygen or free radicals, as anti-microbials, as anti-inflammatory, anti-diabetic, and so on [5]. Phenolic compounds present in plant extract with free hydrogen are primarily responsible for antioxidant activity [72]. Additionally, the significant antioxidant activity might be due to tannins, flavonoids, sugar reduction, polyphenols, and tannins [73].

Acetone, isopropyl alcohol, and ethanol heartwood extracts of *Pterocarpus marsupium* demonstrated excellent antioxidant activity in the radical scavenging system 2,2-diphenyl-1-picrylhydrazyl (DPPH) [74]. Methanol extract (100 μg/mL) of *P*. *marsupium* has the highest radical scavenging effect of 2,2-diphenyl-1-picrylhydrazyl free radical followed by ethyl acetate and aqueous extracts. The scavenging effect reached saturation with extract concentration being further increased. This study showed substantial antioxidant activity in DPPH, superoxide, ABTS, hydroxyl radical, nitric oxide scavenging, and in vitro lipid peroxidation inhibition by *P*. *marsupium* heartwood extract [52]. *Solanum trilobatum* leaf extract synthesized MgO-NPs have a slightly higher DPPH scavenging ability with IC50 72.24 μg mL^−1^ relative to ascorbic acid (IC50 33.46 μg mL^−1^). Sushma et al. [75] and Dobrucka et al. [58] reported antioxidant behavior of *Clitoria ternatea* leaf extract-mediated synthesis of MgO-NPs. The microbially synthesized MgO-NPs showed the maximum free radicals inhibition relative to the ones synthesized utilizing soil and chemical methods.

### Minimum inhibitory concentration

Polyphenols and phenolics present in plants have been known to be harmful to micro-organisms [76]. *Dichrostachys cinerea* root bark tannin has antibacterial activity against *Staphylococcus aureus*, *Escherichia coli*, and *Psuedomonas aeruginosa* [77]. In vitro studies by Chung et al. [78] showed that tannins from different structures prevented the growth of the microorganism. Flavonoids have been documented as having both antibacterial and antifungal activity [79]. Bijase et al. [80] have reported that isoflavonoids are isolated from the methanolic extract of root bark and stem bark of *Bolusanthus specious* exhibits antibacterial activity. Biologically active tannin glycosides, alkaloids, steroids, and flavonoids were found to contain the bark extract [81]. Among the majority of phytoconstituents that have potent antibacterial activity [82, 83], alkaloids also exhibit microbicidal action.

The suspension of phytoassisted magnesium oxide nanoparticles has probably shown significant antibacterial activity against *E*. *coli* and *S*. *aureus* because the MgO-NPs can easily reach the nucleus of the bacteria and provide an excellent surface area for interactions that impede development. Previous studies have reported the efficacy of MgO-NPs and other metallic nanoparticles on antibacterial behavior on the occurrence of oxygen vacancies on the surface of the nanoparticles, which contributed to lipid peroxidation and production of reactive oxygen species [72, 73]. The antibacterial activity of the MgO-NPs against *E*. *coli* was also considered comparable with the silver/ MgO nanocomposites produced by using *Musa paradisiaca* extract [84]. The lipid peroxidation and reactive oxidative species production processes in the container have been responsible for the antibacterial ability of MgO-NPs [85]. Increased pH and Mg2^+^ ions have been predicted to play an important role in the mode of action of MgO nanoparticles against microbes, provided that MgO nanoparticles dissociate in microbial culture and release OH-ions and Mg2^+^ ions [86].

Initial studies of metallic nanoparticles on their antimicrobial activities showed huge potential in the food industry, biomedical science, and many other science and technology sectors because these substances can offer enduring antibacterial activities due to their intrinsic instability and high-temperature tolerance properties [33]. Metallic nano component alternatives are important to significantly increase the magnitude and incidence of multidrug-resistant bacterial strains [87]. In an earlier study by Patil and Gaikwad [86], the *P*. *marsupium* stem bark (heartwood) methanolic extract showed the highest inhibitory effect against *Pseudomonas aeruginosa* followed by *Bacillus subtili*, *S*. *aureus*, *Salmonella typhi*, *E*. *coli*, *Klebsiella pneumoniae*, *Micrococcus* and *Proteus mirabilis*.

*S*. *trilobatum*-mediated MgO-NPs demonstrated significant antibacterial action against *E*. *coli*. Equally, Che-MgO-NPs exhibited maximal inhibition zone found in *E*. *coli* (15.16 ± 0.44 mm) and total inhibitory zone in *Streptococcus pyogenes* (13.83 ± 0.92 mm) at a concentration of 100 mg mL^−1^ [69]. Doss et al. [88] demonstrated that MgO-NPs have antibacterial action against *E*. *coli* which was caused by a wide range of oxygen found on the magnesium surface. Doss et al. [88] and Krishnamoorthy et al. [89] described the increased antibacterial activity of magnesium NPs due to ROS and lipid peroxide with an oxygen defect present on particle morphology. MgO-NPs, strong in electrostatic association with bacterial surface, contributed to cell death [90, 91].

### Alpha-amylase inhibitory activity of MgO-NPs

Thus, it is revealed from inhibitory studies that the *Pterocarpus marsupium*-synthesized MgO nanoparticles are capable of inhibiting the alpha-amylase enzyme, which is additionally beneficial in delaying the break-down of starch into glucose, thereby controlling glucose rates in patients with diabetics. Jeevanandam [92] reported that MgO nanoparticles in diabetes mellitus can revert insulin resistance and be a strong antidiabetic agent. The ethanol extract of heartwood (1 g/kg oral and 2 g/kg oral) greatly decreased the elevated rates of glucose in Wistar male albino rats with antidiabetic results in hyperglycemia and hyperinsulinemia triggered by dexamethasone [93]. Marsupsin and Pterostilbene are the *Pterocarpus marsupium* heartwood’s most effective phenolic compounds after intraperitoneal administration using 40 mg/kg b.wt for each dosage. The blood glucose level in hyperglycemic rats with hyperglycemia caused by streptozotocin, which is mainly useful in non-insulin-dependent diabetes mellitus (NIDDM) with obesity, decreased significantly aligned metformin as control [94].

Aqueous extract of *Pterocarpus marsupium* displayed a significant effect on reducing cytokine TNF-α tested on streptozotocin-induced rats. Cytokine TNF-α was initially elevated in uncontrolled diabetic rats owing to chronic systemic inflammation. Both doses of aqueous extract (100 and 200 mg/kg b.wt.) appreciably reduced the increased TNF-α level in rats due to the isoflavone components in plant aqueous extract [95].

### Effect on protein inhibition

*P*. *marsupium* aqueous extract was observed to reduce elevated inflammatory cytokine, TNF-α rates in NIDDM diabetic rats at doses of 100 mg/kg and 200 mg/kg b.wt [52]. Methanol and aqueous extract of *P*. *marsupium* used carrageenan-mediated rat paw edema approach to test anti-inflammatory activity by the model of acute inflammation. The dosage of methanol extract 50 mg/kg b.wt. and dosage of aqueous extract of 100 mg/kg b.wt. reported a significant decline in paw edema. Both extracts were found to have significant anti-inflammatory activity [96]. Nano-MgO showed stronger analgesic and anti-inflammatory activities in mice at experimental formalin and acetic acid tests [97].

## Conclusion

In the present study, we have reported phytoassisted synthesis of magnesium-oxide nanoparticles using *Pterocarpus marsupium* heartwood extract. The organic molecules in the aqueous extract act as reducing and stabilizing agents. The MgO-NPs have been characterized using UV, DLS, FT-IR, XRD, SEM, and TEM. Magnesium oxide nanoparticles formation was confirmed by a sharp absorption peak at 310 nm. FT-IR analysis proves the formation of MgO nanoparticles by the functional groups of *P*. *marsupium* that act as reducing and stabilizing agents. The average size (16.76 nm) of MgO-NP, which was determined from the Debye Scherer formula, was found by XRD analysis. The observed MgO-NPs is spherically shaped with nanometer size is confirmed in SEM and TEM. EDS analysis has identified the chemical composition of MgO nanoparticles. The antioxidant activity of MgO-NPs was evaluated using DPPH scavenging activity and the IC_50_ value of the MgO-NPs was found to be 89.67 μg/mL. The antibacterial activity of MgO-NPs was calculated by using minimum inhibitory concentration assay against Gram-positive and Gram-negative bacteria and the MIC values obtained are 22 ± 0.168 and 24 ± 0.439 μg/mL. The antidiabetic activity of phytoassisted MgO-NPs was tested on inhibition of α-amylase; the IC_50_ was found to be 56.32. The anti-inflammatory activity of MgO-NPs was tested by the albumin denaturation method; the IC_50_ value was found to be 81.69. Thus magnesium oxide nanoparticles synthesized from *P*. *marsupium* obtained may have possible biomedical applications showing excellent antioxidant, antibacterial, anti-diabetic, and anti-inflammatory properties.

## Data Availability

Not applicable.

## Abbreviations

PM: *Pterocarpus marsupium*
MgO-NPs: Magnesium oxide nanoparticles
MgO: Magnesium oxide
NPs: Nanoparticles
UV-Vis: UV-Visible spectrophotometry
PDI: Polydispersity index
XRD: X-ray diffraction
FT-IR: Fourier transform infrared spectroscopy
SEM: Scanning electron microscopy
EDS: Energy-dispersive X-ray spectroscopy
TEM: Transmission electron microscopy
DLS: Dynamic light scattering
SAED: Selected area electron diffraction
IC_50_: Half-maximal inhibitory concentration
